# Will future microbots be task-specific customized machines or multi-purpose “all in one” vehicles?

**DOI:** 10.1038/s41467-021-26675-0

**Published:** 2021-12-08

**Authors:** Joseph Wang

**Affiliations:** Department of Nanoengineering, University of California San Diego, La Jolla, CA 92093 USA

**Keywords:** Nanoscience and technology, Nanoscale devices

## Abstract

While existing microbots display effective propulsion, their functionalities decrease dramatically upon decreasing the robot size. Accordingly, it is desired to customize microscale robots for their specific mission and body location. Selecting the microbot constituents with task-specific tailored functionalities will enhance their practicality in performing their primary mission.

Over the past 15 years the field of microbots has exploded with many teams from around the globe contributing to major innovations^1–3^. A myriad of synthetic microbots, based on various propulsion mechanisms and different designs and materials, have thus been developed^4–7^. New functionalities and capabilities have been added to these tiny machines, including fast motion in complex biological media, large cargo-towing force, collective behavior and excellent biocompatibility for use in living systems. These attractive capabilities have paved the way to sophisticated microscale robotic devices capable of performing complex tasks and have motivated researchers to explore exciting new important in vivo biomedical applications ranging from targeted drug delivery to precise surgery and intracellular biosensing^8–10^. While the scaling down of robotic platforms has tremendous potential for advancing the treatment and diagnosis of patients, it also brings fundamental engineering challenges such as power sourcing, motion control and tracking, integration of multifunctionality, safety and related recovery and degradation capabilities^11^. Unlike large robots, the tiny footprint of microbots greatly hampers the ability to integrate multiple functions (without compromising their dimensions). For example, important microbot operations, such as drug delivery and cleaning out clogged arteries, have completely different requirements and hence rely on largely incompatible functions, which are extremely challenging to combine with a single microscale robot.

## Simplicity versus complexity: special-purpose or general-purpose microbots?

**Fig. 1:**
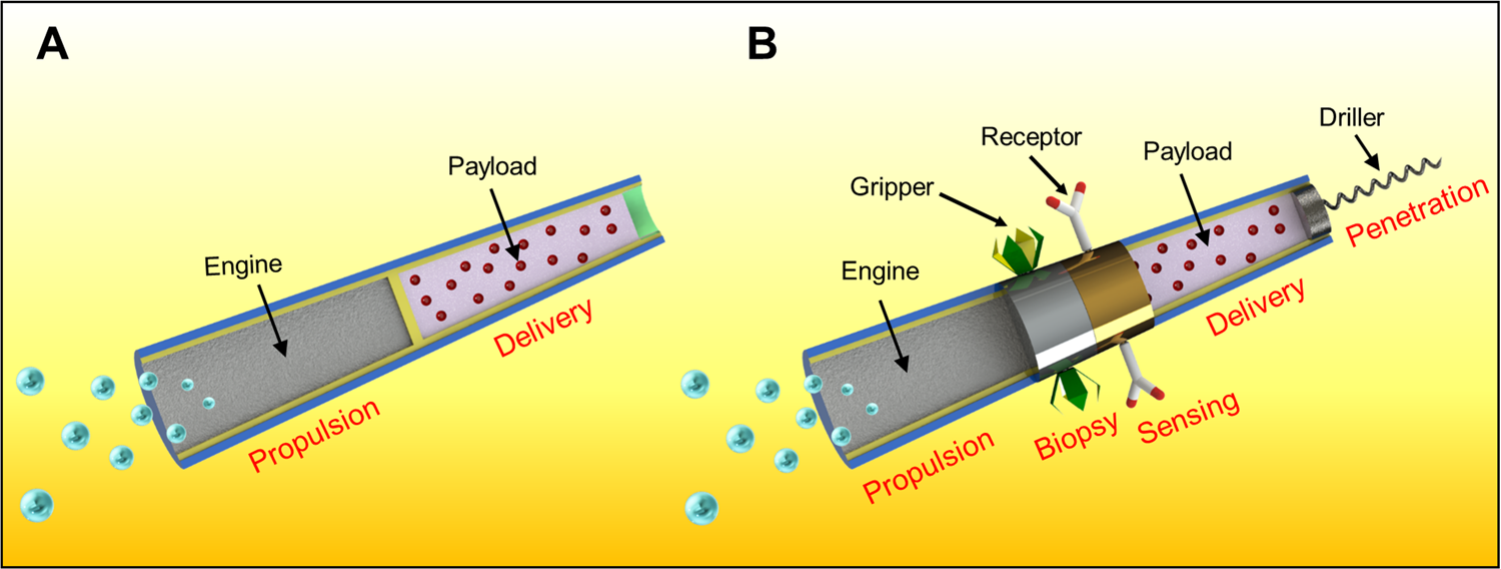
Strategies in designing microbots. Single-purpose customized nanovehicles (**A**) versus multi-purpose “all-in-one” devices (**B**) with different sections used for imparting different functions (delivery, biopsy, sensing and drilling) along with the basic motion requirement.

Considering the major challenges of fabricating multifunctional microscale robots, a key question arises: is the future hold for general-purpose universal microbots capable of performing a wide range of tasks or to simple microbots tailored to meet the demands of specific applications and target locations (Fig. 1)? Big robots commonly excel at specific tasks performed repetitively in structured environments. While such single-task robots have been the initial focus of the robot industry, recent efforts have shifted to general-purpose robots. Similarly, the majority of microscale robots developed to date have been designed to perform single function (Fig. 1A), e.g., drug delivery or biopsy gripping, and hence lack the adaptability and capabilities required to perform different tasks under the diverse conditions of different body locations. More advanced microrobots, combining multiple functions, will be created to execute more complex biomedical operations requiring multiple tasks (Fig. 1B). Similar to a Swiss Army Knife, the design of such multifunctional (multi-tasking) microbots would offer the advantage of combining lot of extra features in one device. However, in most cases, many of these extra functions may be of limited use considering the main mission (which is—in the case of a knife—to cut objects). Eliminating additional features, that are not essential for the primary mission of a microbot, would greatly simplify its design and maximize its efficacy to meet the specific requirements of its main mission. The microbots will thus be custom designed and created based on their usage scenario and the conditions of the targeted body site. For example, an important question facing the selection of the microbot engine: should it be universal for diverse body locations or tailored to a specific site? Chemically powered micromotors can be designed according to the availability of the local fuels, such urea in the bladder or acid in the stomach. In contrast, magnetically actuated microbots are universal and can operate in numerous locations. The efficiency of microbots to perform their primary mission will be maximized using simple robot design tailored made only according to this mission. Such single-purpose microbots are particularly desired considering the remarkably small dimensions of these robots. Independent of their propulsion mode or their primary mission, biomedical microbot must consist of biocompatible and biodegradable material, ensuring their self-destruction when no longer needed.

In contrast, the preparation of multifunctional microscale robots requires a paradigm shift from the traditional mechanical assembly fabrication route used for preparing larger (millimeter) scale robots, e.g., ingestible capsules. While these larger robots commonly rely on different sections to obtain their different functions, microbots are too small to be prepared by such mechanical assembly fabrication route. Some efforts have been devoted to advanced rolled-up and template deposition techniques for fabricating microbots with several functional units^2^, but these fabrication methods also face critical miniaturization constraints, which limit their functional capabilities to few basic functions. Instead, future multifunctional microscale robots could rely on smart multi-responsive materials capable of imparting several mission-specific functions into a single portion of such a microbot for executing multiple tasks. Such bio-inspired responsive materials can enable programmable, reconfigurable mobile microscale robots and increase the adaptability of such microbots for complex operations without scaling up the robot’s footprint^12^. For example, smart biomarker responsive materials can lead to theranostic microbots, performing “sense and release” functions by detecting disease-specific marker to trigger autonomous release of their embedded therapeutic payload^13^. Such route can enable diabetes theranostic applications based on autonomous closed-loop glucose-responsive insulin-delivery gated acoustic vehicles^14^.

## Conclusions

Advanced robotics fabrication and functionalization technologies are paving the way to new robotic capabilities which are attractive for a wide range of biomedical applications. However, despite of these tremendous technological advances, it is extremely challenging to design and efficiently manufacture multifunctional microbots capable of performing diverse tasks. Considering the tiny dimensions of microbots and the largely different operational requirements of different microbot applications and body locations, the design of small-scale multi-purpose robots faces distinct challenges. Typical strategies for preparing large multi-purpose robots cannot be adapted to the microscale.

The efficiency of microbots to perform their main mission will be greatly improved by using simple robot design custom-made specifically to this mission. Designing the microscale robots with task-specific tailored materials and functionalities will allow customization of microbots for their primary biomedical mission and will enhance their practicality and efficiency in performing this mission. Incorporating additional features that are not essential for this specific biomedical operation can greatly compromise their effectiveness to perform their main task. Complex biomedical operations, requiring multiple tasks, will require multifunctional microbots based on new advanced fabrication techniques. In particular, bio-inspired responsive materials offer tremendous promise for designing theranostic microbots, integrating the sense and release functions. Alternately, it may be possible to combine different single-function microbots in swarms for performing different operational tasks^11^. Despite the major progress towards developing such bio-inspired microbots there is still a long way for the performance of synthetic microbots to compete with the sophistication of nature’s biomotors. A multidisciplinary collaboration in science, engineering and medicine and researchers trained with cross-disciplinary research skills are vital for addressing critical barriers for translating microbots into the clinical practice toward establishing a new era in the treatment of diseases.
